# Mitochondrial myopathies diagnosed in adulthood: clinico-genetic spectrum and long-term outcomes

**DOI:** 10.1093/braincomms/fcae041

**Published:** 2024-02-14

**Authors:** Grayson Beecher, Ralitza H Gavrilova, Jay Mandrekar, Elie Naddaf

**Affiliations:** Department of Neurology, Mayo Clinic, Rochester, MN 55905, USA; Division of Neurology, Department of Medicine, University of Alberta, Edmonton, Alberta, Canada T6G 2G3; Department of Neurology, Mayo Clinic, Rochester, MN 55905, USA; Department of Clinical Genomics, Mayo Clinic, Rochester, MN 55905, USA; Department of Neurology, Mayo Clinic, Rochester, MN 55905, USA; Department of Neurology, Mayo Clinic, Rochester, MN 55905, USA

**Keywords:** mitochondrial myopathies, MELAS, *POLG*, chronic progressive external ophthalmoplegia, inherited myopathies

## Abstract

Mitochondrial myopathies are frequently recognized in childhood as part of a broader multisystem disorder and often overlooked in adulthood. Herein, we describe the phenotypic and genotypic spectrum and long-term outcomes of mitochondrial myopathies diagnosed in adulthood, focusing on neuromuscular features, electrodiagnostic and myopathological findings and survival. We performed a retrospective chart review of adult patients diagnosed with mitochondrial myopathy at Mayo Clinic (2005–21). We identified 94 patients. Median time from symptom onset to diagnosis was 11 years (interquartile range 4–21 years). Median age at diagnosis was 48 years (32–63 years). Primary genetic defects were identified in mitochondrial DNA in 48 patients (10 with single large deletion, 38 with point mutations) and nuclear DNA in 29. Five patients had multiple mitochondrial DNA deletions or depletion without nuclear DNA variants. Twelve patients had histopathological features of mitochondrial myopathy without molecular diagnosis. The most common phenotypes included multisystem disorder (*n* = 30); mitochondrial encephalomyopathy, lactic acidosis and stroke-like episodes (14); limb myopathy (13); chronic progressive external ophthalmoplegia (12); and chronic progressive external ophthalmoplegia-plus (12). Isolated skeletal muscle manifestations occurred in 27%. Sixty-nine per cent had CNS and 21% had cardiac involvement. Mutations most frequently involved *MT-TL1* (27) and *POLG* (17); however, a wide spectrum of established and novel molecular defects, with overlapping phenotypes, was identified. Electrodiagnostic studies identified myopathy (77%), fibrillation potentials (27%) and axonal peripheral neuropathy (42%, most common with nuclear DNA variants). Among 42 muscle biopsies available, median percentage counts were highest for cytochrome C oxidase negative fibres (5.1%) then ragged blue (1.4%) and ragged red fibres (0.5%). Skeletal muscle weakness was mild and slowly progressive (decline in strength summated score of 0.01/year). Median time to gait assistance was 5.5 years from diagnosis and 17 years from symptom onset. Thirty patients died, with median survival of 33.4 years from symptom onset and 10.9 years from diagnosis. Median age at death was 55 years. Cardiac involvement was associated with increased mortality [hazard ratio 2.36 (1.05, 5.29)]. There was no difference in survival based on genotype or phenotype. Despite the wide phenotypic and genotypic spectrum, mitochondrial myopathies in adults share similar features with slowly progressive limb weakness, contrasting with common multiorgan involvement and high mortality.

## Teaser

Beecher *et al*. describe the clinical, genetic, electrodiagnostic and myopathological features and long-term outcomes in 94 patients diagnosed with mitochondrial myopathy in adulthood. They highlight that despite the wide genotypic and phenotypic spectrum, mitochondrial myopathies in adults share slowly progressive limb weakness, contrasting with common multiorgan involvement and high mortality.

## Graphical Abstract

**Graphical Abstract:**
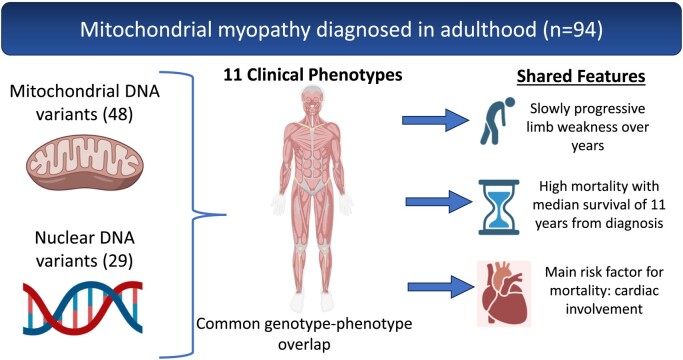

## Introduction

Since the initial descriptions of patients with pathogenic variants in mitochondrial DNA (mtDNA),^1,2^ our understanding of mitochondrial myopathies and their phenotypic, histopathological and genotypic heterogeneity has evolved considerably. Advancements in and increased availability of genetic testing have expanded the mitochondrial disease spectrum.^3^ Mitochondrial myopathies are most commonly recognized in childhood and adolescence,^4^ with specific clinical phenotypes well-established.^5^ However, as recent studies from large nationwide databases have identified,^6-10^ mitochondrial myopathies are frequently associated with diverse clinical features which do not necessarily align with a classical syndrome. Mitochondrial myopathies may be overlooked in adulthood, especially when extraocular muscles (EOM) are spared and the clinical, electrodiagnostic and pathological features across specific clinical and molecular phenotypes in adulthood are less well-characterized. Furthermore, detailed long-term outcomes and survival data among patients with mitochondrial disorders, including mitochondrial myopathies, are scarce.

Herein, we describe the phenotypic and genotypic spectrum of 94 patients diagnosed with mitochondrial myopathy in adulthood at a large referral centre, aiming to correlate clinical and molecular phenotypes with histopathological features, laboratory and electrodiagnostic findings and longitudinal motor outcomes and survival.

## Materials and methods

### Patient selection

The study was performed at the Mayo Clinic (Rochester, Minnesota), which is a tertiary referral centre for neuromuscular and mitochondrial disease. This study was approved by the Mayo Clinic Institutional Review Board (#21–003128). The study was minimal risk; therefore, the requirement for informed consent was waived. However, patients who had not provided authorization for their medical records to be used for research, as per Minnesota statute 144.335, were not reviewed. We searched Mayo Clinic electronic medical records to identify patients with mitochondrial myopathy diagnosed at ≥18 years of age, evaluated at our clinics between 1 January 2005 and 20 April 2021. We included patients having one or more of nuclear DNA (nDNA) pathogenic variants causative of mitochondrial myopathy; mtDNA pathogenic variants: single large-scale or multiple deletions or mtDNA depletion causative of mitochondrial myopathy; and clear histopathological evidence of mitochondrial myopathy on muscle biopsy without molecular aetiology determined despite genetic evaluation or without genetic evaluation performed. Only pathogenic, likely pathogenic or variants of uncertain significance (VUS) highly suspicious to be responsible for the patient’s condition were included.

### Clinical features

Retrospective chart review of patients’ demographic and clinical characteristics was performed. Diagnosis date was defined as the time when mitochondrial myopathy was first established by muscle histopathological evaluation or genetic testing. Patients were categorized by nDNA or mtDNA primary genetic defect and by clinical mitochondrial syndromes as previously described (Supplemental Table 1),^5,6,11-17^ including chronic progressive external ophthalmoplegia (CPEO); CPEO-plus (CPEO+); mitochondrial encephalomyopathy, lactic acidosis and stroke-like episodes (MELAS); sensory ataxic neuropathy, dysarthria and ophthalmoparesis (SANDO); Kearns–Sayre syndrome (KSS); myoclonic epilepsy with ragged red fibres (MERFF); mitochondrial neurogastrointestinal encephalopathy (MNGIE); mitochondrial myopathy with lactic acidosis and sideroblastic anaemia (MLASA); multisystem disorder (clinical manifestations involving three or more organs or tissues, not conforming to a classical syndrome); limb myopathy (affecting limb muscles without PEO); and myopathy affecting limb muscles with additional CNS manifestations (limb myopathy+).

Specific extraskeletal muscle systemic manifestations were documented for each patient including CNS, cardiac, peripheral neuropathy, endocrine, gastrointestinal, respiratory (evidence of neuromuscular respiratory weakness by pulmonary function testing and/or sleep-disordered breathing by overnight oximetry or polysomnogram), constitutional (fatigue, exercise intolerance, BMI < 18.5, short stature), psychiatric, renal and haematologic. Pattern of skeletal muscle weakness was recorded: EOM only, predominantly proximal or distal or both, and EOM plus proximal weakness. Peripheral neuropathy was characterized as clinically apparent (history and/or examination features of peripheral neuropathy) or subclinical (absence of neuropathic symptoms and physical examination findings, with electrodiagnostic studies demonstrating peripheral neuropathy).

We selected outcome measures extractable by chart review: summated strength scores (SSS) and modified Rankin Scale (mRS) scores at first and last visit, time to gait aid and death. Time-based variables were evaluated from symptom onset (per patient’s report) and diagnosis. The SSS included a summated score of bilateral shoulder abduction, elbow flexion, elbow extension, finger flexion, hip flexion, hip abduction, knee extension and ankle dorsiflexion, based on the Mayo Clinic scale: 0 [equivalent to Medical Research Council (MRC) grade 5], −1 (grade 4+), −2 (grade 4), −3 (grade 4–), −3.25 (grade 3), −3.5 (grade 2), −3.75 (grade 1) and −4 (grade 0). SSS score ranges from 0 (no weakness) to −64 (complete paralysis of included muscles). mRS scores were defined as 0, no symptoms; 1, non-disabling symptoms that do not interfere with daily activities; 2, no significant disability despite symptoms (able to carry out all usual duties and activities); 3, moderate disability (requiring some help but able to walk or dress and eat without assistance); 4, moderately severe disability (unable to walk, clothe self without assistance and attend to own bodily needs without assistance); 5, severe disability (bedridden and requiring constant care and attention); and 6, dead.^18^

### Laboratory, muscle histopathology and electrodiagnostic evaluation

Serological studies performed during the diagnostic evaluation were reviewed: resting creatine kinase (CK), lactate, pyruvate and growth differentiation factor-15 (GDF-15) levels. Electrocardiograms, echocardiograms, pulmonary function testing, overnight oximetry, polysomnograms and swallow evaluations (evaluation by a speech pathologist and barium swallow when available) were reviewed. Muscle biopsies were performed at Mayo Clinic as part of the initial diagnostic evaluation. Biopsy sites were chosen on a case-by-base basis by the requesting provider based on clinical examination, in addition to electromyography (EMG) findings when available. Biopsy slides were reviewed by two authors (G.B. and E.N.). Photographs were taken from four low-power field (LPF, 10× magnification; Olympus DP73 microscope camera, cellSens Imaging Software, version 1.18) sections in a standardized fashion to avoid bias, one from each quadrant of the biopsy, and the total number of fibres per section was manually counted and averaged over four sections. The percentage of ragged red fibres (RRF) on modified Gomori trichrome-stained sections, ragged blue fibres (RBF) on succinate dehydrogenase (SDH)-stained sections and cytochrome C oxidase negative (COX−) fibres on COX-stained sections was averaged across four LPFs. The presence of RRF and RBF which were COX− or COX positive (COX+) was recorded. Increased lipid content adjudged by Oil-Red-O-stained sections, strongly SDH-positive blood vessels and muscle fibre denervation atrophy (atrophic, angulated fibres of both histochemical fibre types with increased non-specific esterase reactivity) was recorded. Electrodiagnostic studies (including nerve conduction studies and needle EMG) performed at Mayo Clinic during the diagnostic evaluation were reviewed, with the following recorded: presence and distribution (diffuse, proximal and/or bulbar) of myopathic features (early recruitment of short-duration, low-amplitude motor unit potentials) and the presence of fibrillation potentials, myotonic discharges and peripheral neuropathy.

### Molecular data

Genetic testing reports were reviewed, and nDNA and mtDNA variants, single large-scale or multiple deletions or depletion causative of mitochondrial myopathy were recorded. mDNA sequencing was performed in blood, muscle or both. All genetic tests were performed for clinical purposes in certified clinical laboratories.

### Statistical analysis

Statistical analysis was performed with SAS 9.4 software (SAS Institute Inc., Cary, NC, USA) and BlueSky Statistics 7.10 software (BlueSky Statistics LLC, Chicago, IL, USA). We utilized descriptive summaries: number and percentages for categorical variables and medians/interquartile range (IQR) for continuous variables. We reported data for all patients together and by group (genotype or phenotype). Statistical analysis was performed comparing 2 samples (nDNA versus mtDNA) or 1 group against the others for 11 phenotypes (e.g. CPEO versus others). Patients with multiple mtDNA deletions or mitochondrial depletion syndrome without a known nDNA pathogenic variant were not included in the genotypic subgroup analyses. Fisher exact or Chi-square test compared categorical variables, and Mann-Whitney U or Kruskal–Wallis test compared continuous variables, as appropriate. Time-based outcomes (survival, time to gait assistance) were analysed using the Kaplan–Meier method and Cox proportional hazards regression. We evaluated whether the following variables were predictors of survival (from diagnosis date) in univariable and multivariable analyses: age at symptom onset, age at diagnosis, sex, disease duration, CNS involvement, cardiac involvement, respiratory involvement, dysphagia, type 2 diabetes mellitus and psychiatric involvement. Slopes for MRS and SSS over time were calculated using change from initial to final measurements accounting for the duration of the follow-up, and descriptive summaries were reported. For sensitivity analysis, we performed survival analysis on various predefined classifications to determine whether specific molecular and phenotypic subgroups had more favourable survival based on factors entertained in the literature. We conducted survival analysis by (i) dividing mtDNA defects into single large deletion versus point mutation, (ii) comparing the most common genes identified to the remaining cohort (to determine whether results are mostly driven by variants in *POLG* and *MT-TL1*), (iii) lumping phenotypes: isolated skeletal muscle weakness versus not (as the latter may be expected to have greater frequency of non-skeletal muscle weakness-related complications and possibly mortality) and (iv) three lumped groups: isolated skeletal muscle weakness versus CNS involvement (plus syndromes) versus systemic involvement (to determine whether the presence of isolated skeletal muscle weakness versus CNS involvement or systemic involvement, yielded more favourable survival).

## Results

### Patient demographics

We initially identified 405 adult patients referred for evaluation of possible mitochondrial myopathy. Of those, only 94 met the pre-specified inclusion criteria. Patients were excluded on the basis of an alternative final diagnosis or lack of mtDNA or nDNA pathogenic variant or muscle biopsy that is in keeping with a mitochondrial myopathy. Demographic characteristics are listed in Table 1. Eighty-two patients (87%) had a molecular diagnosis. Twelve (13%) had histopathological features of mitochondrial myopathy without molecular diagnosis achieved, with phenotypes including CPEO (*n* = four), CPEO+ (*n* = one), multisystem disorder (*n* = three) and limb myopathy (*n* = four). The most common phenotypes across the cohort included multisystem disorder (32%), MELAS (15%), limb myopathy (14%), CPEO (13%), CPEO+ (13%) and limb myopathy+ (7%). Eighty-seven patients (93%) were White, four were Black (4%), and three (3%) were Asian. Both sexes were equally affected. The MELAS group had higher percentage of females compared with others (*P* = 0.026). Patients with limb myopathy+ were younger at symptom onset (*P* = 0.044) and diagnosis (*P* = 0.024). Otherwise, age at symptom onset and diagnosis were similar across genotypic and phenotypic subgroups. Delayed diagnosis was common, with median duration from symptom onset to diagnosis of 11 years (IQR: 4–21 years). Sixty-one (65%) patients were diagnosed in the fifth decade or later.

**Table 1:** Demographic, clinical and genetic characteristics of patients by genotypic and phenotypic subgroup at time of diagnosis (*n* = 94)

	Total (*n* = 94)	mtDNA (*n* = 48)	nDNA (*n* = 29)	CPEO (*n* = 12)	CPEO+ (*n* = 12)	Multisystem (*n* = 30)	MELAS (*n* = 14)	Limb myopathy (*n* = 13)	Limb myopathy+ (*n* = 7)	SANDO (*n* = 2)	KSS (*n* = 1)	MERRF (*n* = 1)	MNGIE (*n* = 1)	MLASA (*n* = 1)
Female	48 (51)	27 (56)	11 (38)	5 (42)	5 (42)	16 (53)	11 (79)	4 (31)	4 (57)	1	1	1	1	0
Age at symptom onset (y)	31 (18, 46)	25 (16, 41)	31 (18, 45)	46 (26, 56)	24 (16, 41)	38 (17, 46)	24 (18, 38)	51 (36, 66)	18 (8, 29)	25 (21, 28)	15	22	18	12
Age at diagnosis (y)	48 (32, 63)	43 (29, 56)	41 (30, 56)	56 (50, 58)	53 (41, 61)	55 (36, 54)	38 (26, 52)	64 (38, 67)	30 (22, 44)	37 (35, 38)	26	23	24	40
Myalgia	51 (54)	27 (56)	17 (59)	3 (25)	3 (25)	16 (53)	8 (57)	11 (85)	4 (57)	2	1	1	1	1
Rhabdomyolysis	6 (6)	4 (8)	2 (7)	0 (0)	0 (0)	2 (7)	2 (14)	1 (8)	1 (14)	0	0	0	0	0
Dysphagia	22 (23)	9 (19)	9 (31)	2 (17)	5 (42)	8 (27)	3 (21)	2 (15)	0 (0)	1	0	0	1	0
Extraskeletal muscle manifestations														
CNS	65 (69)	35 (73)	22 (76)	0 (0)	12 (100)	27 (90)	14 (100)	0 (0)	7 (100)	2	1	1	1	0
Peripheral neuropathy	39 (42)	11 (23)	19 (66)	6 (50)	5 (42)	18 (60)	3 (21)	4 (31)	0 (0)	2	0	0	1	0
Cardiac	20 (21)	10 (21)	6 (21)	0 (0)	0 (0)	14 (47)	4 (29)	1 (8)	0 (0)	0	1	0	0	0
Respiratory	31 (33)	15 (31)	10 (35)	7 (58)	3 (25)	12 (40)	2 (14)	4 (31)	2 (29)	1	0	0	0	0
Constitutional	65 (69)	39 (75)	21 (72)	4 (33)	8 (67)	23 (77)	13 (93)	8 (62)	4 (57)	1	1	1	1	1
Psychiatric	21 (22)	10 (21)	9 (31)	1 (8)	2 (17)	9 (30)	4 (29)	3 (23)	1 (14)	1	0	0	0	0
Gastrointestinal	30 (32)	16 (33)	9 (31)	0 (0)	1 (8)	21 (70)	4 (29)	1 (8)	0 (0)	0	1	0	1	1
Endocrine	21 (22)	15 (31)	2 (7)	2 (17)	1 (8)	9 (30)	6 (43)	1 (8)	2 (29)	0	0	0	0	0
Molecular classification														
nDNA variant				4 (33)	7 (58)	11 (37)	0 (0)	1 (8)	2 (29)	2	0	0	1	1
mDNA variant				4 (33)	4 (33)	12 (40)	14 (100)	7 (54)	5 (71)	0	1	1	0	0

Categorical variables are presented as number of patients (percentage), and continuous variables are presented as median (first quartile, third quartile). Percentages are not displayed for groups with one or two patients.

CPEO, chronic progressive external ophthalmoplegia; CPEO+, CPEO-plus; KSS, Kearns–Sayre syndrome; MELAS, mitochondrial encephalomyopathy, lactic acidosis and stroke-like episodes; MERRF, myoclonic epilepsy with RRF; MLASA, mitochondrial myopathy with lactic acidosis and sideroblastic anaemia; MNGIE, mitochondrial neurogastrointestinal encephalopathy; mtDNA, mitochondrial DNA; nDNA, nuclear DNA; SANDO, sensory ataxic neuropathy, dysarthria and ophthalmoparesis.

### Clinical characteristics

Clinical characteristics and extraskeletal muscle manifestations are detailed in Table 1 and Supplemental Table 2. Twenty-five (27%) patients had disease confined to skeletal muscle (CPEO or limb myopathy). Proximal predominant limb weakness was the most common pattern of weakness, occurring in 40 (43%) patients, followed by EOM plus proximal limb in 36 (38%), isolated EOM in 17 (18%) and distal in 1 (1%) (Supplemental Table 2). Patients with primary mtDNA defects most commonly presented with proximal weakness, whereas patients with nDNA defects most commonly presented with EOM plus proximal weakness. Regardless of genotype or phenotype, limb weakness was typically mild, reflected by median SSS at baseline of −2 (Table 2).

**Table 2:** Laboratory features and change in modified Rankin scale and summated strength score over time of mitochondrial myopathies diagnosed in adulthood, by genetic and phenotypic subgroups

	Total	mtDNA	nDNA	CPEO	CPEO+	Multisystem	MELAS	Limb myopathy	Limb myopathy+	SANDO	KSS	MERRF	MNGIE	MLASA
**Laboratory evaluation**														
CK elevated	29/83 (35)	16/44 (36)	9/25 (36)	2/11 (18)	3/8 (38)	9/26 (35)	5/12 (42)	7/13 (54)	1/7 (14)	1/2 (50)	0/1	1/1	0/1	0/1
CK, resting (xULN)	0.8 (0.1–7.0; 83)	0.9 (0.4–1.5; 44)	0.8 (0.4–1.6; 25)	0.8 (0.6–1.0; 11)	1.1 (0.3–1.4; 8)	0.6 (0.3–1.1; 26)	1 (0.1–7; 12)	1.4 (0.5–2.7; 13)	0.7 (0.3–0.9; 7)	1 (0.4–1.6; 2)	0.6	3.2	0.45	0.1
Lactate elevated	35/77 (45)	26/43 (60)	6/22 (27)	3/10 (30)	1/7 (14)	8/24 (33)	12/13 (92)	5/10 (50)	3/7 (43)	0/2 (0)	0/1	1/1	1/1	1/1
Lactate, resting (xULN)	1.0 (0.3–3.2; 77)	1.3 (0.8–1.9; 43)	0.8 (0.7–1.1; 22)	0.6 (0.5–0.6; 10)	0.8 (0.7–1.0; 7)	0.9 (0.6–1.2; 24)	2 (0.8–3.2; 13)	1 (0.7–2.4;10)	0.7 (0.6–1.4; 7)	1 (0.9–1; 2)	0.8	2.4	2.1	1.1
Pyruvate elevated	16/33 (49)	13/19 (68)	2/9 (22)	0/4 (0)	0/1 (0)	4/8 (50)	6/9 (67)	3/5 (60)	1/2 (50)	0/1 (0)	0/1	1/1	1/1	NP
Pyruvate, resting (xULN)	1 (0.4–3; 33)	1.2 (1–1.9; 19)	0.8 (0.6–0.9; 9)	0.8 (0.6–0.8; 4)	0.9 (NA, 1)	1.1 (0.6–1.2; 8)	1.1 (0.9–2.6; 9)	1.5 (0.6–2.2; 5)	0.9 (0.8–0.9; 2)	0.4 (NA, 1)	0.6	1.5	1.1	NP
**mRS and SSS evaluation**														
SSS at baseline	−2 (−7, 0)	−2 (−9, 0)	−2 (−2, −2)	0 (−2, 0)	−1 (−5, 0)	−4 (−10, 0)	−4 (−9, −1)	−2 (−13, 0)	−2 (−1, 0)	−2 (−3, −1)	−5	−5	−2	0
mRS at baseline	2 (2, 2)	2 (2, 2)	2 (2, 2)	2 (2, 2)	2 (2, 2)	2 (2, 2)	2 (2, 3)	2 (2, 2)	1 (1, 2)	2	3	2	3	1
SSS slope	−0.01 (0, −2)	−0.02 (0, −0.53)	0 (0, −0.14)	0 (0, −2)	−0.01 (0, −0.14)	−0.01 (0, −0.53)	−0.04 (0, −0.33)	−0.02, (0, −0.63)	−0.03 (0, −0.08)	−0.01 (0, −0.02)	−0.19	−0.04	0	−0.02
mRS slope	0.02 (0, 4)	0.01 (0, 0.4)	0.02 (0, 4)	0 (0, 0.14)	0.03 (0, 0.1)	0.01 (0, 4)	0.03 (0, 0.3)	0 (0, 0.1)	0.01 (0, 0.04)	0.02 (0.02, 0.02)	0.03	0.01	0	0

Categorical variables are presented as number of patients/number tested (percentage), and continuous variables are presented as median (IQR; number of patients tested). Percentages are not displayed for groups with one patient (KSS, MERRF, MNGIE, MLASA).

CK, creatine kinase; CPEO, chronic progressive external ophthalmoplegia; CPEO+, CPEO-plus; KSS, Kearns–Sayre syndrome; MELAS, mitochondrial encephalomyopathy, lactic acidosis and stroke-like episodes; MERRF, myoclonic epilepsy with RRF; MLASA, mitochondrial myopathy with lactic acidosis and sideroblastic anaemia; MNGIE, mitochondrial neurogastrointestinal encephalopathy; mRS, modified Rankin scale; mtDNA, mitochondrial DNA; NA, not applicable; nDNA, nuclear DNA; NP, not performed; SANDO, sensory ataxic neuropathy, dysarthria and ophthalmoparesis; SSS, strength summated score; ULN, upper limit of normal.

A spectrum of extraskeletal muscle manifestations was identified (Table 1), with CNS (69%), constitutional (69%) and peripheral neuropathy (41%) most frequent. Detailed description is available in Supplemental Table 2. Among 65 patients with CNS involvement, the most common features included sensorineural hearing loss in 42 (45%), migraine in 26 (28%), epilepsy in 22 (23%), encephalopathy or dementia in 18 (19%), stroke-like episodes in 17 (18%) and ataxia in 8 (9%). Patients with MELAS had significantly greater occurrence of migraine, epilepsy, stroke-like episodes, encephalopathy, sensorineural hearing loss, short stature, BMI < 18.5 and diabetes mellitus compared with the cohort (Supplemental Table 2). While stroke-like episodes are classic of MELAS, they occurred in two patients with multisystem disease and one patient with SANDO, all with pathogenic variants in *POLG* (c.1399G>A in homozygosity in two patients and c.1491G>C in compound heterozygosity with c.2243G>C in another). Thirty-nine patients had length-dependent peripheral neuropathy, which was clinically apparent in 34 (87%) and subclinical in 5 (13%). All but two patients had cardiac screening via an ECG with or without transthoracic echocardiogram. Cardiac abnormalities were more common in patients with a multisystem disorder compared with the remainder of the cohort (*P* < 0.0001). CPEO and CPEO+ patients had no cardiac involvement (Supplemental Table 2). Forty-five (48%) patients had screening for respiratory involvement with pulmonary function testing, overnight oximetry or polysomnogram. Respiratory involvement occurred with similar frequency across the cohort, most commonly in multisystem disease and CPEO/CPEO+ (Table 1). Haematologic involvement occurred in two (2%; one MLASA with sideroblastic anaemia, one multisystem with congenital dyserythropoetic anaemia) and renal involvement in one (1%; one multisystem with end-stage renal disease on haemodialysis). Four patients had lipomas (three with mtDNA abnormalities and one patient with MNGIE). One of these four patients with a single large mtDNA deletion had several lower extremities lipomas. Our patient with MERRF had no lipomas.

Amongst genotypic subgroups, extraskeletal manifestations including migraine, short stature and endocrine abnormalities, namely diabetes mellitus, were significantly more common amongst the mtDNA group, while peripheral neuropathy occurred significantly more in the nDNA group (Table 1, Supplemental Table 2).

### Laboratory and electrodiagnostic testing

Laboratory testing results are summarized in Table 2. CK was normal in approximately two-thirds of patients. Lactate and pyruvate levels were more commonly elevated in the mtDNA group than the nDNA group, as was lactate elevation in the MELAS group relative to remaining phenotypic groups. GDF-15 levels were assessed in six patients (two CPEO, one limb myopathy, one limb myopathy+, one multisystem, one MELAS) and elevated in all (median 1965 pg/mL, IQR 1591–2894 pg/mL).

Electrodiagnostic studies were performed at Mayo Clinic in 74 patients (Supplemental Table 3). Nine patients (12%) had normal studies. Myopathic changes occurred in 57 (77%), fibrillation potentials in 20 (27%), myotonic discharges in 4 (5%; 3 with single large mitochondrial deletions, 1 with *POLG*) and axonal sensorimotor peripheral neuropathy in 31 (42%) patients. Electrodiagnostic features were similar among subgroups, although axonal peripheral neuropathy was more common in the nDNA group compared with the mtDNA group (*P* = 0.0007). Facial muscles were infrequently sampled by EMG outside of patients with CPEO/CPEO+ or multisystem disease; however, in patients with CPEO/CPEO+, a high rate (70%) of facial myopathic motor unit potentials was identified, including four patients where myopathic changes were not identified in limb muscles.

### Muscle biopsies

**Figure 1:**
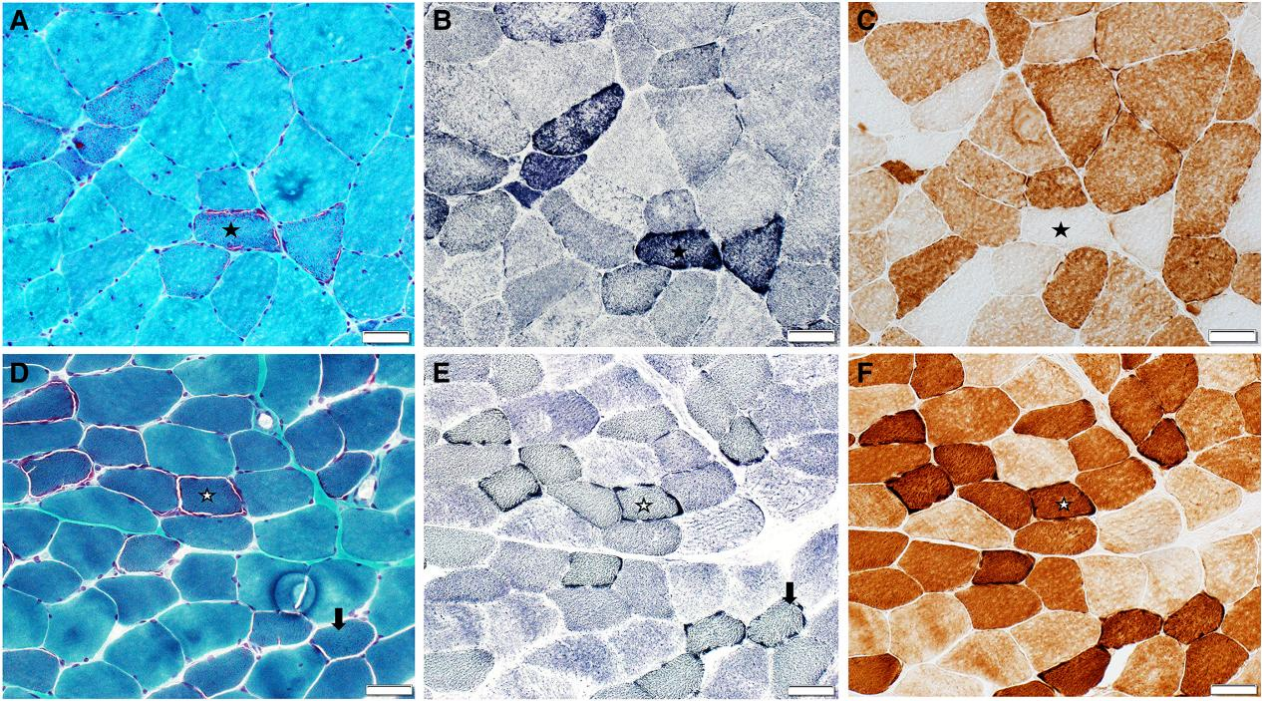
**Muscle biopsy findings.** Serial frozen sections from two representative patients with mitochondrial myopathy, stained with modified Gomori trichrome (**A** and **D**), SDH (**B** and **E**) and CCO (**C** and **F**). Note that each row represents an individual patient. The most common pattern consisted of the presence of (**A**) RRF and (**B**) RBF that are (**C**) CCO negative (example fibre is marked with a star). The less common pattern consisted of (**D**) RRF and (**E**) RBF fibres that are (**F**) CCO positive (example fibre is marked with a star). Overall, CCO-negative fibres as seen in (**C**) were the most commonly encountered mitochondrial abnormality. RBF were more common than RRF. An example of RBFs on SDH (**E**) that are not identifiable as RRF on trichrome sections (**D**) is marked with an arrow. Scale bar: 50 µm.

Muscle biopsies were performed in 62 patients during the diagnostic evaluation. Muscle biopsy sites included quadriceps (23 patients), biceps (13), triceps (9), deltoid (8) and others (9). Forty-two biopsies were available for quantification of mitochondrial abnormalities (Table 3, Fig. 1). COX− fibres were most common, followed by RBFs then RRFs. RRF and RBF were more frequent in the mtDNA group compared with the nDNA group. Significant differences in percentage of RRFs, RBFs or COX− fibres were not identified amongst phenotypic groups or amongst those with either mtDNA point mutations or deletions (data not shown).

**Table 3:** Quantifiable mitochondrial abnormalities in muscle biopsies of patients diagnosed with mitochondrial myopathy in adulthood

	Ragged red fibres (%)	Ragged blue fibres (%)	Cytochrome c oxidase negative fibres (%)
**Total (*n* = 42)**	0.5 (0–1.3)	1.4 (0.2–3.6)	5.1% (3.2–7.0)
**Molecular category**			
mtDNA pathogenic variants (*n* = 16)	0.8 (0.2–2.1)	3 (1.2–5.8)	5.5 (4.4–8.6)
nDNA pathogenic variants (*n* = 11)	0.1 (0–0.5)	0.6 (0.1–2.3)	5.6 (3.1–8)
Molecular aetiology undetermined (*n* = 15)	0.8 (0–8.6)	1.6 (0–8.9)	3.5 (0–13.3)
**Phenotypic category**			
CPEO (*n* = 7)	0.8 (0.3–1.3)	2.2 (1.2–3.0)	4.1 (3.3–8.0)
CPEO+ (*n* = 5)	1.1 (0.8–2.2)	4.6 (1.5–5.9)	12.7 (3.7–13.8)
Multisystem (*n* = 15)	0 (0–0.1)	0.2 (0–1.2)	4.7 (2.7–6.5)
MELAS (*n* = 3)	2.9 (1.4–4.0)	5.8 (3.0–7.9)	5.5 (1.8–8.3)
Limb myopathy (*n* = 9)	0.8 (0.4–1.2)	1.6 (1.2–3.5)	5.2 (4.3–5.8)
Limb myopathy+ (*n* = 1)	1.2 (NA)	3.6 (NA)	0 (NA)
SANDO (*n* = 1)	0 (NA)	0.2 (NA)	5.6 (NA)
KSS (*n* = 1)	0.3 (NA)	2.4 (NA)	4.7 (NA)

Continuous variables are presented as median (first to third quartile).

CPEO, chronic progressive external ophthalmoplegia; CPEO+, CPEO-plus; KSS, Kearns–Sayre syndrome; MELAS, mitochondrial encephalomyopathy, lactic acidosis and stroke-like episodes; MERRF, myoclonic epilepsy with RRF; MLASA, mitochondrial myopathy with lactic acidosis and sideroblastic anaemia; MNGIE, mitochondrial neurogastrointestinal encephalopathy; mtDNA, mitochondrial DNA; NA, not applicable; nDNA, nuclear DNA; SANDO, sensory ataxic neuropathy, dysarthria and ophthalmoparesis.

Patients with nDNA point mutations (8 *POLG*, 1 *OPA1*, 1 *RRM2B*, 1 *YARS2*) commonly displayed scattered COX− fibres in 10 (91%) and RBF/COX− fibres in 7 (70%). Four biopsies (40%) with COX− fibres additionally displayed RBF/COX+ fibres. All 10 patients with single large or multiple mtDNA deletions without a known nDNA variant featured scattered COX− fibres and RBF/COX− fibres; however, three patients additionally demonstrated RBF/COX+ fibres (two with single deletions spanning m.9000_13500 or m.7000_13918 and one with multiple deletions).

Specific mtDNA variants demonstrated unique features. Two patients with pathogenic variants in mtDNA genes encoding respiratory chain subunits other than complex IV had RBF/COX+ fibres (one *MT-CYB* m.15595delC and one *MT-ND5* m.13513G>A, encoding subunits for complexes III and I, respectively). In biopsies from two patients with *MT-TL1* m.3243A>G, COX− fibres were present in both; however, RBFs were solely COX+ in one and predominantly COX+ (with few COX−) in the second. One patient with *MT-TL1* m.3251A>G and one patient with *MT-TI* m.4281A>G had RBFs which were solely COX+, in addition to COX− fibres which were not RBF. A single patient with *MT-TM* m.4412G>A had RBFs which were both COX− and COX+.

SDH-positive blood vessels and abnormal lipid storage were not present in any biopsy. Denervation atrophy occurred in 26 (61%) and did not correlate with age at biopsy. There was no significant correlation between percentage of RRFs, RBFs or COX− fibres with age at biopsy, CK, lactate, pyruvate, SSS, strength of biopsied muscle or mRS at diagnosis.

### Genetic testing and genotype–phenotype correlation

**Figure 2:**
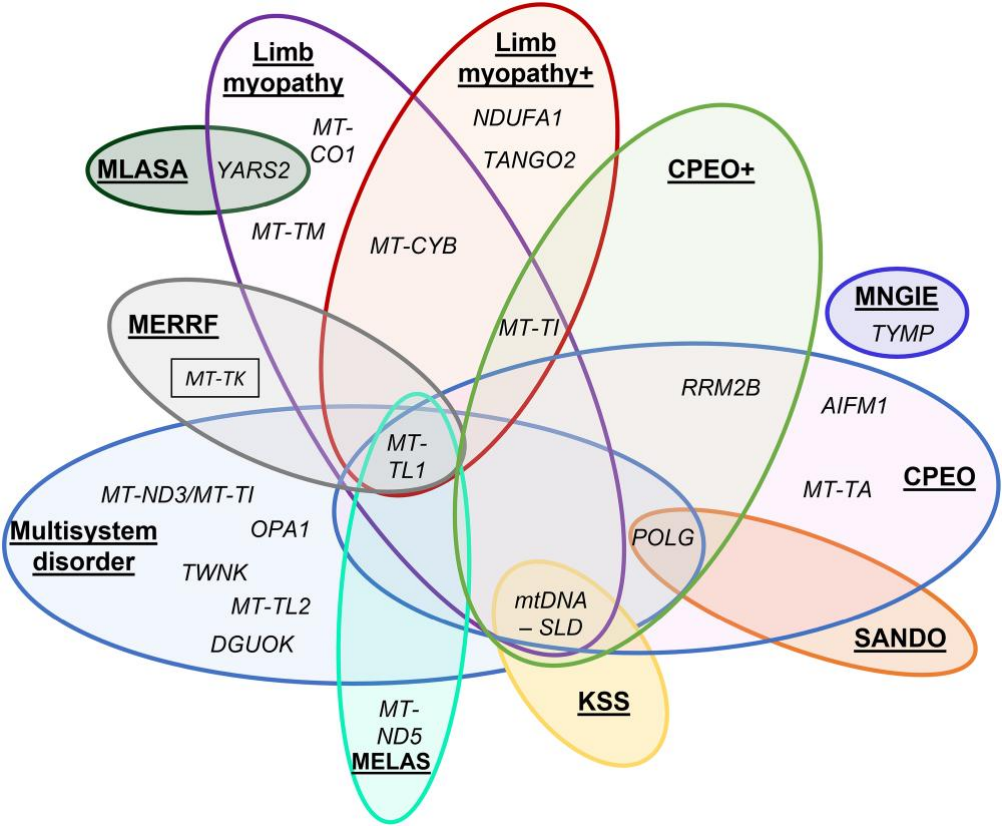
**Genotype–phenotype correlation in mitochondrial myopathies diagnosed in adulthood.** Venn diagram demonstrating the wide variability and overlap between various nDNA and mitochondrial DNA genes and clinical phenotypes in this cohort. Phenotypes represented in the cohort included CPEO, CPEO+, MELAS, SANDO, KSS, MERFF, MNGIE, MLASA, multisystem disorder, limb myopathy and limb myopathy+. The most common MERRF-associated gene described in the literature, not represented in our cohort, is additionally shown in a small black box. CPEO, chronic progressive external ophthalmoplegia; CPEO+, CPEO-plus; KSS, Kearns–Sayre syndrome; limb myopathy+, myopathy affecting limb muscles with additional CNS manifestations; MELAS, mitochondrial encephalomyopathy, lactic acidosis and stroke-like episodes; MERRF, myoclonic epilepsy with RRF; MLASA, mitochondrial myopathy with lactic acidosis and sideroblastic anaemia; MNGIE, mitochondrial neurogastrointestinal encephalopathy; mtDNA, mitochondrial DNA; mtDNA-SLD, mtDNA single large deletion; multisystem, clinical manifestations involving three or more organs or tissues, not conforming to a classical syndrome; nDNA, nuclear DNA; SANDO, sensory ataxic neuropathy, dysarthria and ophthalmoparesis.

Detailed genetic testing information is provided in Supplemental Table 4. Primary mtDNA defects (*n* = 48) were more frequent than nDNA defects (*n* = 29). Ten patients had a single large mtDNA deletion, whereas 38 patients had an mtDNA point mutation. Eight patients had multiple mtDNA deletions, including four with no identifiable nDNA pathogenic variant. One patient had an mtDNA depletion syndrome without identifiable nDNA pathogenic variant. There was major overlap between clinical phenotypes and genotypes (Fig. 2). mtDNA sequencing was performed in blood in 29 patients, muscle in 49 patients and both blood and muscle in 21 patients. Amongst these, heteroplasmy level was higher in muscle (median: 62%; IQR 27.5–73), than blood (median: 24%; IQR: 15.25–42.35) (*P* = 0.009).

Patients with a multisystem disorder had a wide spectrum of underlying genotypes, with most common mutations in *POLG* (eight patients) or *MT-TL1* (seven patients). Among patients with MELAS, 13/14 had a mutation in *MT-TL1* (m.3243A>G in 12 and 1 with m.3251A>G) and 1 in *MT-ND5*. Patients with CPEO/CPEO+ most commonly had mutations in *POLG* (*n* = seven) followed by a single large mtDNA deletion (*n* = five). In patients with a limb myopathy (limb myopathy/limb myopathy+) without PEO, mtDNA single large deletions were uncommon (single patient), and mtDNA point mutations (11/15, 73%) were more common than nDNA point mutations (3/15, 20%). Patients with SANDO, MERRF, MNGIE and MLSA had mutations in *POLG*, *MT-TL1*, *TYMP* and *YARS2*, respectively. Overall, the most commonly involved gene was *MT-TL1* (27 patients). Only 48% of patients with mutations in *MT-TL1* fulfilled criteria for MELAS; however, all patients, except one, had extraskeletal muscle manifestations. The second most commonly involved gene was *POLG* (17 patients), associated with a wide spectrum of clinical manifestations, with all patients having EOM involvement and most (15/17, 88%) with extraskeletal muscle manifestations.

### Longitudinal outcomes

**Figure 3:**
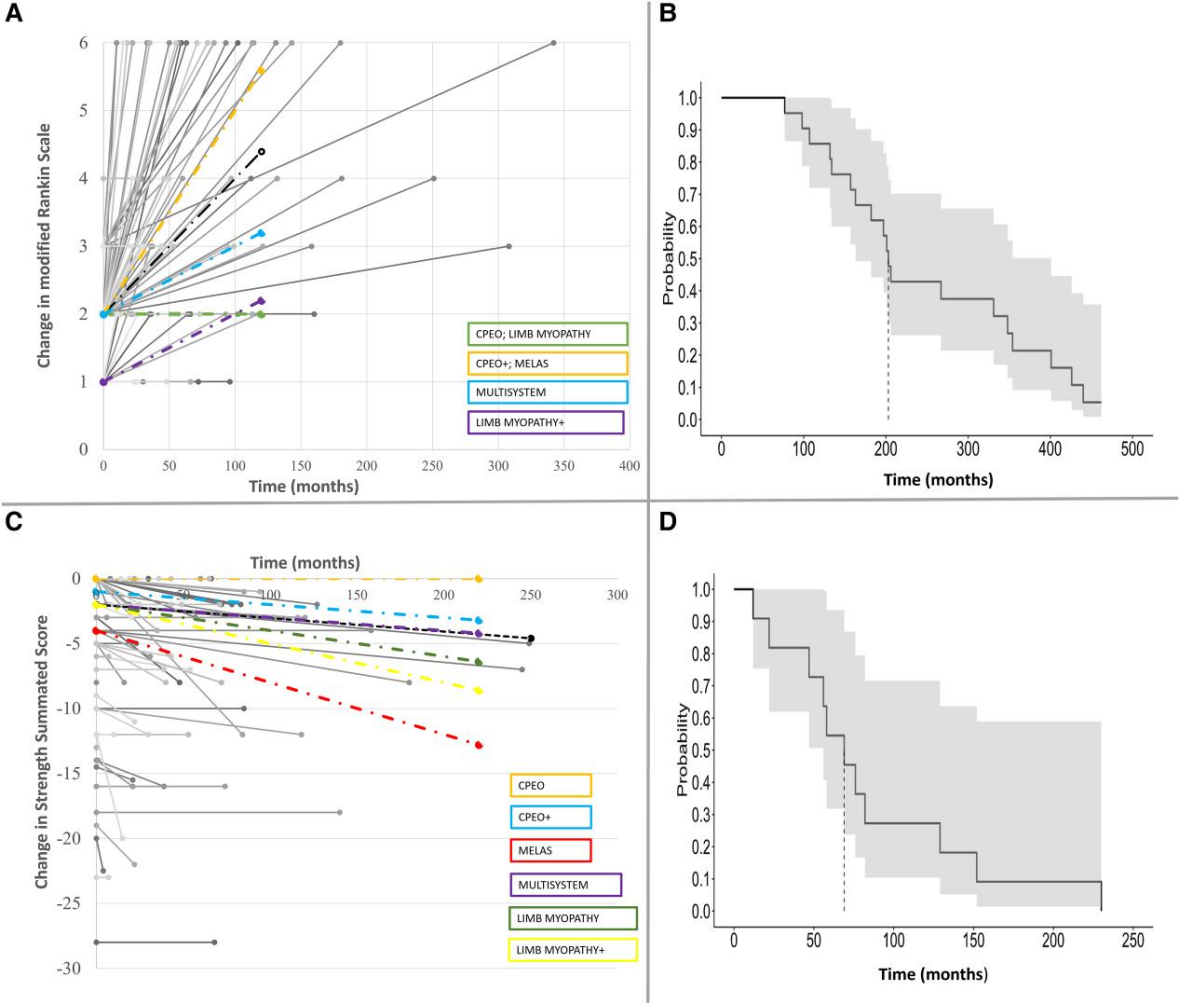
**Motor function and disability in mitochondrial myopathies diagnosed in adulthood.** (**A**) Change in modified Rankin scale over time. Each grey line represents an individual patient’s MRS score at initial presentation and last follow-up. Median linear regression lines for various phenotypes are shown as colour-coded. The black dashed line represents the median linear regression line for the whole cohort. Kaplan–Meier curve, with 95% confidence interval, showing time to gait assistance from (**B**) symptom onset and (**D**) from diagnosis. Median time is shown as a vertical dashed line. (**C**) Change in strength summated score (SSS) over time. Each grey line represents an individual patient’s SSS score at initial presentation and last follow-up. Median linear regression lines for various phenotypes are shown as colour-coded. The black dashed line represents the median linear regression line for the whole cohort. CPEO, chronic progressive external ophthalmoplegia; CPEO+, CPEO-plus; limb myopathy+, myopathy affecting limb muscles with additional CNS manifestations; MELAS, mitochondrial encephalomyopathy, lactic acidosis and stroke-like episodes; multisystem, clinical manifestations involving three or more organs or tissues, not conforming to a classical syndrome.

Median follow-up was 51 months (25–96) from diagnosis and 208 months (112–343) from symptom onset. Follow-up that included a motor examination to calculate an SSS was available for 72 patients, with median follow-up 45.5 months (21–82) from diagnosis. SSS and mRS upon presentation are summarized in Table 2. Change in SSS and mRS over time is demonstrated in Fig. 3, with median slopes of −0.01/year and 0.02/year, respectively. Patients with CPEO demonstrated relatively stable limb weakness (with no limb weakness on average), while other phenotypes, including CPEO+, had slow decline in SSS (Table 2). The fastest decline in SSS occurred in MELAS and limb myopathy+. Similarly, patients with CPEO and limb myopathy demonstrated relatively stable mRS (median of 2), whereas mRS increase was most pronounced in CPEO+ and MELAS. Gait aids were required in 19 patients (20%; 7 multisystem, 4 CPEO+, 2 limb myopathy, 2 limb myopathy+, 2 SANDO, 1 KSS, 1 MELAS). Median time to gait aid (any type) was 69 months from diagnosis and 203 months from symptom onset (Fig. 3). Patients with nDNA defects more commonly required gait aids than those with mtDNA defects (38% versus 13%, respectively, *P* = 0.009).

**Figure 4:**
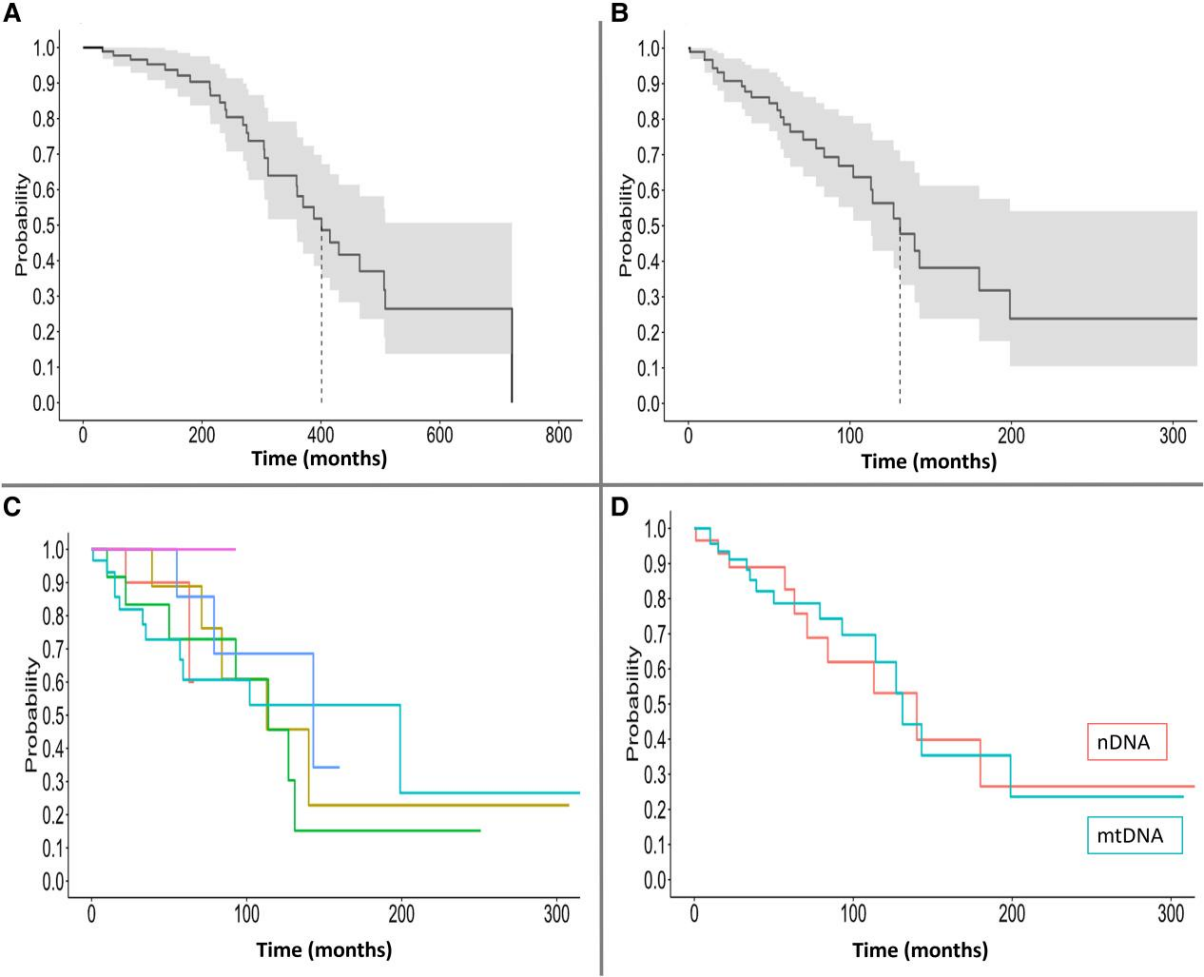
**Survival of patients with mitochondrial myopathies diagnosed in adulthood.** Kaplan–Meier curves showing overall survival from symptom onset (**A**) and from diagnosis (**B**) with median survival shown as a dashed vertical line; comparison of survival from diagnosis among clinical phenotypes [median survival in months: CPEO (*n* = 12), not reached; CPEO+ (*n* = 12), 113; MELAS (*n* = 14), 114; multisystem disorder (*n* = 14), 199; limb myopathy (*n* = 13), 143; limb myopathy+ (*n* = 7), not reached; log-rank *P*-value: 0.74] (**C**); comparison of survival from diagnosis in patients with primary nuclear genetic defect (*n* = 29) versus mitochondrial defect (*n* = 48): median survival of 140 months for primary nuclear versus 131 months for primary mitochondrial defects; log-rank *P*-value = 0.92 (**D**). CPEO, chronic progressive external ophthalmoplegia; CPEO+, CPEO-plus; limb myopathy+, myopathy affecting limb muscles with additional CNS manifestations; MELAS, mitochondrial encephalomyopathy, lactic acidosis and stroke-like episodes; mtDNA, mitochondrial DNA; multisystem, clinical manifestations involving three or more organs or tissues, not conforming to a classical syndrome; nDNA, nuclear DNA; SANDO, sensory ataxic neuropathy, dysarthria and ophthalmoparesis.

Thirty patients (32%; 12 multisystem, 7 MELAS, 5 CPEO+, 3 myopathy, 2 CPEO, 1 SANDO) died during follow-up. Median age at death was 55 years (44.25–69.5). Median survival was 33.4 years from symptom onset and 10.9 years from diagnosis (Fig. 4). Survival was not significantly different between genotypic and phenotypic groups. No patients with limb myopathy+ died during follow-up. Cause of death was unavailable in the medical record in 17 (56%), aspiration pneumonia in 3 (10%; 2 CPEO+, 1 MELAS), sudden unexplained death in epilepsy in 2 (7%; 2 MELAS), cardiac arrest in 2 (7%; 1 SANDO, 1 multisystem disorder), decompensated heart failure in 2 (7%; 2 multisystem disorder), status epilepticus in 1 (3%; 1 MELAS) or other (oesophageal rupture, lung cancer, complications post-bowel obstruction, sepsis) in 4 (13%). Cardiac involvement was the main predictor of mortality in univariable analyses [hazard ratio (HR) 2.36; Supplemental Table 5). Disease duration (at diagnosis) was mildly associated with survival (HR 1.003 per month). HRs remained relatively unchanged in a multivariable model adjusting for cardiac involvement and disease duration (data not shown). Sensitivity analysis comparing patients with single large deletions versus point mutations, *POLG* and *MT-TL1* to the rest of the cohort, isolated skeletal muscle weakness to those without or to those with CNS involvement and those with systemic involvement yielded no significant differences in survival (data not shown).

## Discussion

In this study, we show that mitochondrial myopathies diagnosed in adulthood manifest a wide spectrum of overlapping phenotypes and genotypes. However, they all share similar features consisting of very slowly progressive skeletal muscle weakness contrasting with multiorgan involvement and high mortality, with cardiac involvement being an independent risk factor of mortality. This series also highlights that while EOM involvement usually helps raise suspicion for a mitochondrial disorder, a large proportion of patients present with isolated limb weakness (43% in our series). In those patients, mtDNA mutations were more common and would have been missed if only nuclear genes were sequenced (e.g. myopathy gene panel, whole exome sequencing). No patient presented with isolated fatigue or exercise intolerance. Similar to previous reports, heteroplasmy levels were also higher in muscle tissue compared with blood.^19,20^

There is limited literature on the natural history and long-term functional outcomes of patients with mitochondrial myopathies diagnosed in adulthood. In our cohort, limb muscle strength was relatively stable and declined very slowly over the years, while mRS increased in a clinically significant fashion, mostly driven by mortality (mRS 6) with potential contributors outside skeletal muscle weakness. Patients with isolated skeletal muscle weakness (CPEO and limb myopathy) had a relatively stable mRS over time. Recent efforts to prospectively characterize functional outcomes in an Italian cohort of 112 adult patients with primary mitochondrial myopathies similarly identified stable motor outcomes at 12 months demonstrated via 6-min walk test (6MWT), timed up-and-go test × 3 (3TUG) and five-times sit-to-stand test (5XSST), while there was significant decline in a global assessment of function, the Newcastle Mitochondrial Disease Scale for Adults (NMDAS).^21^ These findings highlight the need to utilize outcome measures with content beyond motor function alone, to validate commonly used outcome measures in mitochondrial myopathies and to develop new disease-tailored measures. Despite utilizing different outcome measures to Montano *et al*.’s cohort, additional subgroup similarities exist between the studies. Patients with CPEO in our series had a relatively stable course compared with other phenotypes, and Montano *et al*.^21^ identified that patients with CPEO had better longitudinal performance on 6MWT, 3TUG, 5XSST and NMDAS compared with those with CPEO and limb weakness and those with mitochondrial myopathies without EOM involvement. Our study adds to the Montano *et al*. study by identifying slowly progressive decline in motor function across multiple phenotypic subgroups over a longer period of follow-up (median 45.5 months), relatively greater decline in patients with MELAS and limb myopathy+, frequent need for gait aids, and expanding our understanding of long-term survival across phenotypes.

Despite mild differences in motor strength and disability between genotypic and phenotypic groups, survival was relatively similar among all groups with high mortality (median survival 11 years from diagnosis) and median age of death of 55 years. Prior retrospective studies of adults with mitochondrial disease, including but not limited to those with myopathy, reported mortality rates ranging from 8% (median follow-up unavailable) to 23% (median follow-up 8.9 years) and relatively similar median age at death of 50–51 years.^22,23^ Annual incidence of death is estimated at 2.5%.^23^ Similar to Barends *et al*.’s^22^ study, causes of death available in the medical record in our cohort were limited with approximately half lacking a documented aetiology; however, complications of epilepsy, cardiac disease and swallowing dysfunction were most common. Aspiration pneumonia leading to death in two patients with CPEO+ aligns with relatively high rates of dysphagia in this group^24-26^ and highlights the need for regular monitoring of swallowing in CPEO/CPEO+. Cardiac involvement was the main independent predictor of death in our cohort, consistent with results from prior retrospective studies of mortality in adulthood^23^ and paediatric mitochondrial myopathy.^27^ Early and ongoing cardiac surveillance is important for identification and timely treatment of cardiac dysfunction in mitochondrial myopathy. Lastly, disease duration was mildly associated with increased mortality, highlighting the importance of developing new disease-modifying therapies.

Percentage quantification of mitochondrial abnormalities in muscle biopsy, including RRF, RBF and COX− fibres, has been performed with varied methodology in smaller series mainly encompassing patients with mtDNA point mutations or deletions.^28-36^ Percentage cut-off criteria for diagnosis of mitochondrial myopathy are proposed, but not validated, and include >2% RRF and/or >2% COX− fibres for an individual <50 years old, 1–2% RRF for an individual aged 30–50 years old, >5% COX-deficient fibres for an individual >50 years old or any RRF in an individual <30 old.^37^ Here, we find COX− fibres more frequent than RBF and RBF more frequent than RRF across the cohort, consistent with trends previously described.^29-32,35,36^ Notably, our median %RRF (0.5%) is below the above-suggested cut-off values for patients older than 30, while our median %COX− fibres (5.1%) are just above the cut-off value for those older than 50; some patients lacked any RRF or COX− fibres, and 11 (26%) biopsies would not have met proposed criteria. Despite the proposed cut-off values, the lack of RRF or COX− fibres on biopsy does not exclude mitochondrial myopathy in the appropriate clinical context. Evaluation for COX− fibres is, however, likely most sensitive for the detection of mitochondrial abnormalities except in patients with variants in mtDNA genes encoding respiratory chain subunits other than complex IV (*MT-CYB* and *MT-ND5* in this series),^38,39^ where predominantly COX+ fibres, some of which correspond to RBFs, are instead found. COX+ RBF fibres are not unique to variants affecting non-complex IV respiratory chain subunits and feature prominently in our patients with variants in mtDNA genes involved in protein synthesis including *MT-TL1*, *MT-TI* and *MT-TM*, as previously described,^5,40^ and may be seen in conjunction with a mosaic pattern of COX− RBFs in patients with single large mtDNA deletions. In contrast to previous work describing increasing disease severity with increasing COX− percentages,^33^ we did not find an association between disease severity as measured by strength of biopsied muscle, SSS and mRS and %RRF, %RBF or %COX− fibres at the time of biopsy.

Dedicated cross-sectional evaluation of needle EMG findings in patients with mitochondrial myopathy is limited.^41-45^ EMG in mitochondrial myopathy is frequently reported as normal or myopathic, typically without fibrillation potentials.^46^ Here, we identified a high rate of electrodiagnostic evidence of myopathy (77%), greater than the previous high (58%) reported by Girlanda *et al*.^43^ We also identify high yield of facial muscle sampling for the detection of myopathy in CPEO/CPEO+, even in the absence of myopathic motor unit changes in limb muscles. This is important given that many patients with CPEO/CPEO+ are reported with normal EMG despite their myopathic clinical phenotype.^24,44^ Fibrillation potentials were identified in just over one-quarter of our cohort, indicating the characterization of mitochondrial myopathy as one occurring only rarely with fibrillation potentials may be overemphasized. Petty and colleagues^41^ described features of denervation in 12% (11/61) of patients with mitochondrial myopathy; otherwise, quantification of fibrillation potential occurrence has not been performed in large cohorts. Myotonic discharges, described twice previously in mitochondrial myopathy due to single large^44^ or multiple deletions in mtDNA,^47^ were observed here in four patients and support the addition of mitochondrial myopathy (particularly with mtDNA deletions) to the growing list of myopathies associated with myotonic discharges.^48^

There is growing recognition that electrodiagnostic evaluation in mitochondrial myopathy can both confirm the presence and identify subclinical evidence of peripheral neuropathy.^44,49,50^ Our findings of clinically apparent or subclinical neuropathy in 41% support this assertion and affirm the significantly greater occurrence of neuropathy in patients with nDNA pathogenic variants.^51,52^ We also identify a high rate of age-independent denervation atrophy (61%) similar to the rate (67%) observed in a recent pathological study of muscle biopsies from 58 patients with mitochondrial myopathy,^53^ again supporting co-occurrence of a neuropathic process in many patients.

This cohort of patients also highlights challenges that permeate the diagnosis of mitochondrial disorders, as on average diagnosis was established 11 years after symptom onset. Notably, the rarity of mitochondrial disease and the significant clinical, molecular, electrodiagnostic and myopathological heterogeneity across patients, even amongst those with the same molecular diagnosis, can contribute to diagnostic uncertainty. As a result, patients frequently seek out the opinion of multiple specialists, undergo numerous investigations and can face lengthy delays before the ultimate diagnosis of a mitochondrial disorder.^54^ While several diagnostic biomarkers, such as lactate, GDF15, FGF21, amino acids, acylcarnitines, organic acids and respiratory chain complex activity measurement by spectrophotometry, may help raise suspicion for a mitochondrial myopathy, a molecular diagnosis is required to establish the diagnosis with certainty.^55,56^ However, genetic testing is still not widely accessible, especially given the high cost of mitochondrial genome sequencing.

Our study has limitations, including those inherent to a retrospective study design. Most patients included in this study were White, limiting generalizability to other races. However, this is relatively similar to the general population seen at our centre and to the racial distribution observed in a large North American cohort of mitochondrial myopathies across the age spectrum.^6^ Muscle biopsy sites were chosen by providers on a case-by-case basis and were not uniform. Muscle biopsies were not available for review from patients with MERRF, MNGIE or MLASA; therefore, myopathological findings may not be applicable to these groups. Although the 12 patients that were diagnosed with mitochondrial myopathy based on histopathological findings had highly suggestive clinical phenotypes and/or severe mitochondrial dysfunction on muscle biopsy, the diagnosis could not be 100% confirmed without an identified genetic defect. A pervasive limitation in mitochondrial myopathy studies includes the clinical heterogeneity of the disease, the challenge of appropriate phenotypic subgrouping and whether a ‘lumping’ or ‘splitting’ approach should be applied.^5^ Here, we have attempted to evaluate from both perspectives, grouping patients by molecular and phenotypic categories with the aim to provide insight across broader molecular and specific phenotypic subgroups; however, we acknowledge that differing classification schema are employed across different studies and consistency in classification is variable. However, we demonstrated that survival is relatively homogenous, regardless of the adopted classification.

## Conclusion

Despite the wide phenotypic and genotypic spectrum, mitochondrial myopathies diagnosed in adults share similar features with very slowly progressive limb weakness, contrasting with common multiorgan involvement and high mortality. Timely diagnosis is of utmost importance to monitor for and manage potentially lethal complications. There is a critical need to develop disease-modifying therapies for patients with mitochondrial myopathy.

## Supplementary material

Supplementary material is available at *Brain Communications* online.

## Supplementary Material

fcae041_Supplementary_Data

## Data Availability

Anonymized data not published within this article will be made available upon reasonable request from any qualified investigator.
