# Supplementary material for: Mitochondrial myopathies diagnosed in adulthood: clinico-genetic spectrum and long-term outcomes
Source: Brain Commun. 2024 Feb 14;6(2):fcae041. doi: 10.1093/braincomms/fcae041 (PMC10906953; doi:10.1093/braincomms/fcae041)
Supplement: Supplementary file 1 [file fcae041_supplementary_data.zip › Supplementary_material.odt]

Supplementary Table 1. Clinical phenotypic classification criteria of mitochondrial myopathies

| Phenotypic category | Criteria |
| --- | --- |
| CPEO1 | Progressive external ophthalmoplegia, ptosis or both +/- limb weakness, dysphagia |
| CPEO+1 | The following must be present: Progressive external ophthalmoplegia, ptosis or both CNS involvement +/- limb weakness, dysphagia |
| MELAS2 | The following three criteria must be present: Stroke-like episodes before age 40 years Encephalopathy characterized by seizures and/or dementia Mitochondrial myopathy is evident by the presence of lactic acidosis and/or ragged-red fibers on muscle biopsy. And two or more of the following criteria are present: Normal early psychomotor development Recurrent headaches Recurrent vomiting episodes |
| Multisystem3 | Involvement of 3 or more organ systems not conforming to a classical clinical phenotype |
| Limb myopathy | Skeletal myopathy affecting limb muscles without progressive external ophthalmoplegia |
| Limb myopathy+ | The following must be present: Skeletal myopathy affecting limb muscles without progressive external ophthalmoplegia CNS involvement |
| SANDO4 | The following must be present: Sensory ataxia Peripheral neuropathy Dysarthria Ophthalmoparesis |
| KSS5 | The following must be present: Onset before age 20 Progressive external ophthalmoplegia Pigmentary retinopathy And at least one of the following must be present: Complete heart block CSF protein greater than 100mg/dL Cerebellar syndrome |
| MERRF6 | The following must be present: Myoclonic epilepsy Ataxia Myopathy |
| MNGIE7 | The following must be present: Progressive external ophthalmoplegia Ptosis Gastrointestinal dysmotility Cachexia Peripheral neuropathy Leukoencephalopathy with increased T2-weighted signal in brain white matter |
| MLASA8 | The following must be present: Myopathy Lactic acidosis Sideroblastic anemia |

CPEO, chronic progressive external ophthalmoplegia; KSS, Kearns-Sayre syndrome; MELAS, mitochondrial encephalomyopathy, lactic acidosis, stroke-like episodes; MERRF, myoclonic epilepsy with ragged red fibers; MLASA, mitochondrial myopathy with lactic acidosis and sideroblastic anemia; MNGIE, mitochondrial neurogastrointestinal encephalopathy; SANDO, sensory ataxic neuropathy with dysarthria and ophthalmoparesis.

Supplementary References for Supplementary Table 1:

1. Hirano M, Akman HO, DiMauro S. Metabolic and Mitochondrial Myopathies in Adults In: Louis ED, Mayer SA, Noble JM, eds. Merritt's Neurology 14th ed. Lippincott Williams & Wilkins; 2022:1011-1018.

2. Hirano M, Ricci E, Koenigsberger MR, et al. Melas: an original case and clinical criteria for diagnosis. Neuromuscul Disord. 1992;2(2):125-35. doi:10.1016/0960-8966(92)90045-8

3. Barca E, Long Y, Cooley V, et al. Mitochondrial diseases in North America: An analysis of the NAMDC Registry. Neurol Genet. Apr 2020;6(2):e402. doi:10.1212/nxg.0000000000000402

4. Fadic R, Russell JA, Vedanarayanan VV, Lehar M, Kuncl RW, Johns DR. Sensory ataxic neuropathy as the presenting feature of a novel mitochondrial disease. Neurology. 1997;49(1):239-245. doi:10.1212/wnl.49.1.239

5. Moraes CT, DiMauro S, Zeviani M, et al. Mitochondrial DNA deletions in progressive external ophthalmoplegia and Kearns-Sayre syndrome. N Engl J Med. May 18 1989;320(20):1293-9. doi:10.1056/NEJM198905183202001

6. Finsterer J, Zarrouk-Mahjoub S, Shoffner JM. MERRF Classification: Implications for Diagnosis and Clinical Trials. Pediatr Neurol. 2018;80:8-23. doi:10.1016/j.pediatrneurol.2017.12.005

7. Hirano M. Mitochondrial Neurogastrointestinal Encephalopathy Disease. In: Adam MP, Mirzaa GM, Pagon RA, et al, eds. GeneReviews((R)). 1993.

8. Riley LG, Cooper S, Hickey P, et al. Mutation of the mitochondrial tyrosyl-tRNA synthetase gene, YARS2, causes myopathy, lactic acidosis, and sideroblastic anemia--MLASA syndrome. Am J Hum Genet. 2010;87(1):52-59. doi:10.1016/j.ajhg.2010.06.001

Supplementary Table 2. Pattern of weakness and extraskeletal muscle manifestations in mitochondrial myopathies diagnosed in adulthood

|  | Total (n=94) | mtDNA (n=48) | nDNA (n=29) | CPEO (n=12) | CPEO+ (n=12) | Multisystem (n=30) | MELAS (n=14) | Myopathy (n=13) | Myopathy+ (n=7) | SANDO (n=2) | KSS (n=1) | MERRF (n=1) | MNGIE (n=1) | MLASA (n=1) |
| --- | --- | --- | --- | --- | --- | --- | --- | --- | --- | --- | --- | --- | --- | --- |
| Pattern of weakness |
| Extraocular | 17 (18) | 4 (8) | 7 (24) | 7 (58) | 8 (67) | 1 (3) | 0 (0) | 0 (0) | 0 (0) | 1 | 0 | 0 | 0 | 0 |
| Proximal | 40 (43) | 31 (65) | 3 (10) | 0 (0) | 0 (0) | 9 (30) | 10 (71) | 12 (92) | 7 (100) | 0 | 0 | 1 | 0 | 1 |
| Distal | 1 (1) | 0 (0) | 1 (3) | 0 (0) | 0 (0) | 0 (0) | 0 (0) | 1 (8) | 0 (0) | 0 | 0 | 0 | 0 | 0 |
| Extraocular + proximal | 36 (38) | 13 (27) | 18 (62) | 5 (42) | 4 (33) | 20 (67) | 4 (29) | 0 (0) | 0 (0) | 1 | 1 | 0 | 1 | 0 |
| Central Nervous System Involvement |
| Epilepsy | 22 (23) | 15 (31) | 7 (24) | 0 (0) | 2 (17) | 6 (30) | 10 (71) | 0 (0) | 1 (14) | 1 | 1 | 1 | 0 | 0 |
| Stroke-like episodes | 17 (18) | 14 (29) | 3 (10) | 0 (0) | 0 (0) | 2 (7) | 14 (100) | 0 (0) | 0 (0) | 1 | 0 | 0 | 0 | 0 |
| Migraine | 26 (28) | 19 (40) | 5 (17) | 0 (0) | 1 (8) | 11 (26) | 9 (64) | 0 (0) | 5 (71) | 0 | 0 | 0 | 0 | 0 |
| Dystonia | 3 (3) | 0 (0) | 3 (10) | 0 (0) | 0 (0) | 1 (3) | 0 (0) | 0 (0) | 2 (29) | 0 | 0 | 0 | 0 | 0 |
| Parkinsonism | 3 (3) | 2 (4) | 1 (3) | 0 (0) | 1 (8) | 1 (4) | 0 (0) | 0 (0) | 0 (0) | 0 | 0 | 1 | 0 | 0 |
| Tremor | 6 (6) | 0 (0) | 6 (21) | 0 (0) | 3 (25) | 2 (7) | 0 (0) | 0 (0) | 0 (0) | 1 | 0 | 0 | 0 | 0 |
| Ataxia | 8 (9) | 2 (4) | 5 (17) | 0 (0) | 3 (25) | 2 (7) | 0 (0) | 0 (0) | 0 (0) | 2 | 0 | 1 | 0 | 0 |
| Encephalopathy/ dementia | 18 (19) | 10 (21) | 6 (21) | 0 (0) | 2 (17) | 5 (17) | 8 (57) | 0 (0) | 2 (29) | 0 | 0 | 0 | 1 | 0 |
| Retinitis pigmentosa | 6 (6) | 6 (13) | 0 (0) | 0 (0) | 1 (8) | 2 (7) | 1 (7) | 0 (0) | 1 (14) | 0 | 1 | 0 | 0 | 0 |
| Optic nerve atrophy | 4 (4) | 1 (2) | 2 (7) | 0 (0) | 1 (8) | 2 (7) | 0 (0) | 0 (0) | 0 (0) | 1 | 0 | 0 | 0 | 0 |
| Sensorineural hearing loss | 42 (45) | 24 (50) | 11 (38) | 0 (0) | 7 (58) | 18 (60) | 10 (71) | 0 (0) | 6 (86) | 1 | 0 | 0 | 0 | 0 |
| Constitutional Involvement |
| Short stature | 14 (15) | 14 (29) | 0 (0) | 0 (0) | 0 (0) | 4 (13) | 7 (50) | 0 (0) | 2 (29) | 0 | 0 | 1 | 0 | 0 |
| BMI<18.5 | 13 (14) | 10 (21) | 3 (10) | 0 (0) | 1 (8) | 4 (13) | 6 (43) | 1 (8) | 0 (0) | 0 | 0 | 0 | 1 | 0 |
| Fatigue | 62 (66) | 39 (81) | 19 (66) | 4 (33) | 8 (67) | 21 (70) | 12 (86) | 8 (62) | 4 (57) | 1 | 1 | 1 | 1 | 1 |
| Exercise intolerance | 24 (26) | 18 (38) | 6 (21) | 4 (33) | 3 (25) | 5 (17) | 5 (36) | 3 (23) | 2 (29) | 0 | 1 | 1 | 0 | 0 |
| Endocrine Involvement |
| Diabetes mellitus | 17 (18) | 13 (25) | 1 (3) | 1 (8) | 0 (0) | 7 (23) | 6 (43) | 1 (8) | 2 (29) | 0 | 0 | 1 | 0 | 0 |
| Hypothyroidism | 6 (6) | 3 (6) | 1 (3) | 1 (8) | 1 (8) | 3 (10) | 0 (0) | 1 (8) | 0 (0) | 0 | 0 | 0 | 1 | 0 |
| Hypogonadism | 2 (2) | 1 (2) | 1 (3) | 0 (0) | 0 (0) | 2 (7) | 0 (0) | 0 (0) | 0 (0) | 0 | 0 | 0 | 0 | 0 |
| Cardiac Involvement |
| Cardiac conduction block | 8 (9) | 5 (10) | 3 (10) | 0 (0) | 0 (0) | 6 (20) | 0 (0) | 1 (8) | 0 (0) | 0 | 1 | 0 | 0 | 0 |
| Structural abnormalities* | 11 (12) | 8 (17) | 3 (10) | 0 (0) | 0 (0) | 7 (23) | 4 (29) | 0 (0) | 0 (0) | 0 | 0 | 0 | 0 | 0 |
| Wolff-Parkinson-White syndrome | 1 (1) | 1 (2) | 0 (0) | 0 (0) | 0 (0) | 1 (3) | 0 (0) | 0 (0) | 0 (0) | 0 | 0 | 0 | 0 | 0 |
| Lipoma | 4 (4) | 3 (6) | 1 (3) | 1 (8) | 0 (0) | 1 (3) | 0 (0) | 1 (8) | 0 (0) | 0 | 0 | 0 | 1 | 0 |

Note: Categorical variables are presented as number of patients (percentage). Percentages are not displayed for groups with 1 or 2 patients. CPEO, chronic progressive external ophthalmoplegia; KSS, Kearns-Sayre syndrome; MELAS, mitochondrial encephalomyopathy, lactic acidosis, stroke-like episodes; MERRF, myoclonic epilepsy with ragged red fibers; MLASA, mitochondrial myopathy with lactic acidosis and sideroblastic anemia; MNGIE, mitochondrial neurogastrointestinal encephalopathy; mtDNA, mitochondrial DNA; nDNA, nuclear DNA; SANDO, sensory ataxic neuropathy with dysarthria and ophthalmoparesis. *Includes non-ischemic cardiomyopathy (dilated, hypertrophic), right/left ventricular hypertrophy, left atrial enlargement.

Supplementary Table 3. Electrodiagnostic study findings in mitochondrial myopathies diagnosed in adulthood by genotypic and phenotypic subgroups

|  | Total (n=74) | mtDNA (n=36) | nDNA (n=23) | CPEO (n=11) | CPEO+ (n=8) | Multisystem (n=25) | MELAS (n=10) | Limb Myopathy (n=11) | Limb Myopathy+ (n=5) | SANDO (n=2) | KSS (n=1) | MNGIE (n=1) |
| --- | --- | --- | --- | --- | --- | --- | --- | --- | --- | --- | --- | --- |
| Diffuse myopathy | 20 (27) | 10 (28) | 6 (26) | 4 (36) | 1 (13) | 4 (16) | 4 (40) | 5 (46) | 2 (40) | 0 | 0 | 0 |
| Proximal myopathy | 33 (45) | 18 (50) | 8 (35) | 3 (27) | 1 (13) | 15 (60) | 4 (40) | 5 (46) | 3 (60) | 2 | 0 | 0 |
| Bulbar myopathy | 15 (60) | 3 (50) | 9 (69) | 5 (100) | 2 (40) | 7 (58) | 0 (0) | 0 (0) | NP | 1 | NP | NP |
| Fibrillation potentials | 20 (27) | 10 (28) | 5 (22) | 1 (9) | 1 (13) | 7 (28) | 5 (50) | 6 (55) | 0 (0) | 0 | 0 | 0 |
| Myotonic discharges | 4 (5) | 3 (8) | 1 (4) | 0 (0) | 1 (13) | 2 (8) | 0 (0) | 1 (9) | 0 (0) | 0 | 0 | 0 |
| Axonal peripheral neuropathy | 31 (42) | 9 (25) | 16 (70) | 5 (46) | 3 (38) | 14 (56) | 3 (30) | 3 (27) | 0 (0) | 2 | 0 | 1 |

Note: Categorical variables are presented as number of patients (percentage). Percentages are not displayed for groups with 1 or 2 patients. Facial muscles were sampled in 25 patients across the following groups: mtDNA (6), nDNA (13), CPEO (5), CPEO+ (5), multisystem (12), MELAS (1), Limb Myopathy (1), SANDO (1). *1 p=0.0007; CPEO, chronic progressive external ophthalmoplegia; KSS, Kearns-Sayre syndrome; MELAS, mitochondrial encephalomyopathy, lactic acidosis, stroke-like episodes; MERRF, myoclonic epilepsy with ragged red fibers; MLASA, mitochondrial myopathy with lactic acidosis and sideroblastic anemia; MNGIE, mitochondrial neurogastrointestinal encephalopathy; mtDNA, mitochondrial DNA; nDNA, nuclear DNA; NP, not performed; SANDO, sensory ataxic neuropathy with dysarthria and ophthalmoparesis.

Supplementary Table 4. Distribution of nDNA and mtDNA abnormalities by clinical phenotype

| Patient | Phenotypic Group | nDNA gene | Allele 1 | Allele 2 | mtDNA abnormality | mtDNA gene | mtDNA variant or deleted region |
| --- | --- | --- | --- | --- | --- | --- | --- |
| 1 | CPEO | NP | NP | NP | NP | NP | NP |
| 2 | CPEO | AIFM1 | c.710A>C | NA | NA | NA | NA |
| 3 | CPEO | NA | NA | NA | Single large deletion |  | m.8482_13447 |
| 4 | CPEO | POLG | c.1399G>A | c.752C>T and c.1760C>T | NA | NA | NA |
| 5 | CPEO | NP | NP | NP | NP | NP | NP |
| 6 | CPEO | NA | NA | NA | NP | NP | NP |
| 7 | CPEO | NP | NP | NP | Single large deletion |  | m.9000_13500 |
| 8 | CPEO | NA | NA | NA | Point mutation | MT-TA | m.5650C>T |
| 91 | CPEO | POLG | c.3287G>A | c.2601T>C | NA | NA | NA |
| 10 | CPEO | RRM2B | c.979C>T | NA | NA | NA | NA |
| 11 | CPEO | NP | NP | NP | Point mutation | MT-TL1 | m.3243A>G |
| 12 | CPEO | NA | NA | NA | NP | NP | NP |
| 13 | CPEO+ | NA | NA | NA | Single large deletion |  | 7.7 kb deletion |
| 14 | CPEO+ | NA | NA | NA | Single large deletion |  | m.8483_13459 |
| 15 | CPEO+ | NA | NA | NA | NA | NA | NA |
| 162 | CPEO+ | POLG | c.2542G>A | c.3104+3A>T | NA | NA | NA |
| 17 | CPEO+ | POLG | c.1399G>A | c.1399G>A | NA | NA | NA |
| 18# | CPEO+ | POLG | c.1491G>C | c.2243G>C | NP | NP | NP |
| 19 | CPEO+ | NP | NP | NP | Point mutation | MT-TI | m.4264G>Aa |
| 20 | CPEO+ | RRM2B | c.671T>G | NA | Multiple deletions |  |  |
| 21 | CPEO+ | NP | NP | NP | Single large deletion |  | m.6024_14815 |
| 22 | CPEO+ | POLG | c.2070+1 G>A | c.2243 G>C | NP | NP | NP |
| 23 | CPEO+ | POLG | c.1399G>A | c.2243G>C | NP | NP | NP |
| 24 | CPEO+ | RRM2B | c.979C>T | NA | NA | NA | NA |
| 25 | MELAS | NP | NP | NP | Point mutation | MT-TL1 | m.3243A>G |
| 26 | MELAS | NA | NA | NA | Point mutation | MT-TL1 | m.3243A>G |
| 27 | MELAS | NA | NA | NA | Point mutation | MT-TL1 | m.3243A>G |
| 28 | MELAS | NA | NA | NA | Point mutation | MT-TL1 | m.3243A>G |
| 29* | MELAS | POLG | c.2704A>C | NA | Point mutation | MT-TL1 | m.3251A>G |
| 30 | MELAS | NP | NP | NP | Point mutation | MT-TL1 | m.3243A>G |
| 31 | MELAS | NP | NP | NP | Point mutation | MT-TL1 | m.3243A>G |
| 32 | MELAS | NP | NP | NP | Point mutation | MT-TL1 | m.3243A>G |
| 33 | MELAS | NP | NP | NP | Point mutation | MT-TL1 | m.3243A>G |
| 34 | MELAS | NA | NA | NA | Point mutation | MT-ND5 | m.13513G>A |
| 35 | MELAS | NP | NP | NP | Point mutation | MT-TL1 | m.3243A>G |
| 36* | MELAS | POLG | c.2704A>C | NA | Point mutation | MT-TL1 | m.3251A>G |
| 37 | MELAS | NP | NP | NP | Point mutation | MT-TL1 | m.3243A>G |
| 38 | MELAS | NP | NP | NP | Point mutation | MT-TL1 | m.3243A>G |
| 39 | Multisystem | NA | NA | NA | NA | NA | NA |
| 40 | Multisystem | NA | NA | NA | Multiple deletions |  |  |
| 413 | Multisystem | NP | NP | NP | Point mutation | MT-TL1 | m.3255G>A |
| 424 | Multisystem | OPA1 | c.113_130delb | NA | Multiple deletions |  |  |
| 43 | Multisystem | NP | NP | NP | Point mutation | MT-TL1 | m.3243A>G |
| 44 | Multisystem | POLG | c.1887C>Gc | NA | NP | NP | NP |
| 45 | Multisystem | POLG | c.1399G>A | c.1399G>A | NA | NA | NA |
| 46 | Multisystem | POLG | c.1399G>A | c.1399G>A | NA | NA | NA |
| 47 | Multisystem | NA | NA | NA | Single large deletion |  | m.5794_14876 |
| 48 | Multisystem | NA | NA | NA | Point mutation | MT-TL2 | m.12318G>A |
| 49 | Multisystem | POLG | c.1399G>A | c.3412C>T | NP | NP | NP |
| 50 | Multisystem | NP | NP | NP | Point mutation | MT-TL1 | m.3243A>G |
| 51 | Multisystem | POLG | c.2243G>C | c.2857C>T | NP | NP | NP |
| 522 | Multisystem | POLG | c.2245T>C | c.3104+3A>T | Multiple deletions |  |  |
| 53 | Multisystem | POLG | c.2828G>A | NA | NP | NP | NP |
| 54 | Multisystem | NA | NA | NA | Depletion (19% of control copy number) |  |  |
| 55 | Multisystem | NA | NA | NA | NA | NA | NA |
| 56# | Multisystem | POLG | c.1491G>C and c.2243G>C | c.1491G>C and c.2243G>C | NA | NA | NA |
| 57 | Multisystem | NA | NA | NA | Point mutation | MT-ND3 and MT-TI | m.10158T>Ad and m.4295A>Ge |
| 585 | Multisystem | TWINKLE | c.1364T>C | NA | NA | NA | NA |
| 59 | Multisystem | NP | NP | NP | NA | NA | NA |
| 60 | Multisystem | NP | NP | NP | Point mutation | MT-TL1 | m.3243A>G |
| 61 | Multisystem | NA | NA | NA | Multiple deletions |  |  |
| 62 | Multisystem | NA | NA | NA | Point mutation | MT-TL1 | m.1606G>A |
| 63 | Multisystem | NA | NA | NA | Multiple deletions |  |  |
| 64 | Multisystem | NA | NA | NA | Single large deletion |  | m.3578_15546 |
| 65 | Multisystem | DGUOK | c.462T>A | del exon 2 | NA | NA | NA |
| 66 | Multisystem | NA | NA | NA | Point mutation | MT-TL1 | m.3243A>G |
| 67 | Multisystem | NA | NA | NA | Single large deletion |  | m.7500_13989 |
| 68 | Multisystem | NP | NP | NP | Point mutation | MT-TL1 | m.3243A>G |
| 69 | Myopathy | NA | NA | NA | Point mutation | MT-CO1 | m.7222A>G |
| 70 | Myopathy | NP | NP | NP | NP | NP | NP |
| 71 | Myopathy | NA | NA | NA | Point mutation | MT-CO1 | m.6145G>A |
| 72 | Myopathy | NA | NA | NA | NA | NA | NA |
| 73 | Myopathy | NA | NA | NA | NA | NA | NA |
| 74 | Myopathy | NA | NA | NA | Point mutation | MT-TM | m.4412G>A |
| 75 | Myopathy | NA | NA | NA | Point mutation | MT-CYB | m.15761G>A |
| 766 | Myopathy | NA | NA | NA | Point mutation | MT-TI | m.4281A>Gf |
| 77 | Myopathy | NA | NA | NA | Point mutation | MT-TL1 | m.3243A>G |
| 78 | Myopathy | NA | NA | NA | Multiple deletions |  |  |
| 79 | Myopathy | NP | NP | NP | NP | NP | NP |
| 80 | Myopathy | YARS2 | c.201dupT | NAg | NA | NA | NA |
| 81 | Myopathy | NP | NP | NP | Single large deletion |  | m.7000_13918 |
| 82 | Myopathy+ | NA | NA | NA | Point mutation | MT-TL1 | m.3243A>G |
| 83 | Myopathy+ | NA | NA | NA | Point mutation | MT-TL1 | m.3243A>G |
| 84 | Myopathy+ | NDUFA1 | c.94G>C | NA | NA | NA | NA |
| 85 | Myopathy+ | TANGO2 | del exons 3-9 | del exons 3-9 | NA | NA | NA |
| 86 | Myopathy+ | NP | NP | NP | Point mutation | MT-TL1 | m.3243A>G |
| 87 | Myopathy+ | NP | NP | NP | Point mutation | MT-TL1 | m.3243A>G |
| 88 | Myopathy+ | NP | NP | NP | Point mutation | MT-CYB | m.15595delC |
| 89 | KSS | NP | NP | NP | Single large deletion |  | m.9499_13723 |
| 90 | SANDO | POLG | c.1399G>A | c.1399G>A | NA | NA | NA |
| 91 | SANDO | POLG | c.1399G>A | c.1399G>A | NA | NA | NA |
| 927 | MLASA | YARS2 | c.359dupG | c.751A>Gh | NP | NP | NP |
| 93 | MERRF | NP | NP | NP | Point mutation | MT-TL1 | m.3256C>T |
| 94 | MNGIE | TYMP | c.1048C>T | c.1048C>T | NP | NP | NP |

Patients from the same family are marked with the same symbol-superscript (#, *). Variants of uncertain significance (VUS) are marked with a number-superscript with a comment below. NA, testing performed and no abnormality identified; NP, testing not performed.

Comments on VUS:

aMitochondrial myopathy on muscle biopsy; novel variant, changes G:C pairing to an A:C mis-pairing in the acceptor stem of tRNA Ile (ClinVar allele ID: 677709); 71% heteroplasmy in muscle

bConflicting evidence regarding pathogenicity. ClinVar allele ID: 210979

cNot present in population databases, segregated with phenotype in 3 family members, missense variants in nearby residues reported pathogenic.

dm.10158T>A is not part of a known haplotype and m.1015T>C is known pathogenic

em.4295 is part of a known haplogroup but also reported in affected individuals and demonstrated in functional studies with reduced complex III activity

fAdressed in reference 6

gNo second mutations was found in YARS2. Patient had lactic acidosis but no anemia. 24% residual YARS2 activity in fibroblasts.

hPatient had mitochondrial myopathy, lactic acidosis and sideroblastic anemia.

Supplementary References for Supplementary Table 4: Previously reported patients.

1. Paul P, Alhammad R, Naddaf E. Clinical Reasoning: Bilateral ptosis, dysphagia, and progressive weakness in a patient of French-Canadian background. Neurology. 2020;95(20):933-938. 

2. Milone M, Wang J, Liewluck T, Chen LC, Leavitt JA, Wong LJ. Novel POLG splice site mutation and optic atrophy. Arch Neurol. 2011;68(6):806-811.

3. Nishigaki Y, Tadesse S, Bonilla E, et al. A novel mitochondrial tRNA(Leu(UUR)) mutation in a patient with features of MERRF and Kearns-Sayre syndrome. Neuromuscul Disord. 2003;13(4):334-340.

4. Milone M, Younge BR, Wang J, Zhang S, Wong LJ. Mitochondrial disorder with OPA1 mutation lacking optic atrophy. Mitochondrion. 2009;9(4):279-281.

5. Milone M, Klassen BT, Landsverk ML, Haas RH, Wong LJ. Orthostatic tremor, progressive external ophthalmoplegia, and Twinkle. JAMA Neurol. 2013;70(11):1429-1431.

6. Emmanuele V, Sotiriou E, Shirazi M, et al. Recurrent myoglobinuria in a sporadic patient with a novel mitochondrial DNA tRNA(Ile) mutation. J Neurol Sci. 2011;303(1-2):39-42.

7. Lazaridis KN, Schahl KA, Cousin MA, et al. Outcome of Whole Exome Sequencing for Diagnostic Odyssey Cases of an Individualized Medicine Clinic: The Mayo Clinic Experience. Mayo Clin Proc. 2016;91(3):297-307.

Supplementary Table 5: Cox-proportional hazard ratios evaluating factors associated with mortality in mitochondrial myopathies diagnosed in adulthood

| Variable | Hazard Ratio | 95% CI | p-value |
| --- | --- | --- | --- |
| Age at symptom onset | 1 | 0.98-1.03 | 0.97 |
| Age at diagnosis | 1.02 | 1-1.05 | 0.07 |
| Sex (male) | 2.10 | 0.96-4.61 | 0.06 |
| Disease duration (symptom onset to diagnosis in months) | 1.003 | 1.001-1.006 | 0.018 |
| CNS involvement | 1.37 | 0.518-3.646 | 0.52 |
| Cardiac involvement | 2.36 | 1.05-5.29 | 0.0374 |
| Psychiatric involvement | 1.24 | 0.53-2.88 | 0.62 |
| Dysphagia | 0.92 | 0.40-2.12 | 0.85 |
| Diabetes mellitus | 1.56 | 0.568-3.59 | 0.30 |
| Respiratory involvement | 0.71 | 0.31-1.64 | 0.41 |
